# Fas ligand mediates immune privilege and not inflammation in human colon cancer, irrespective of TGF-*β* expression

**DOI:** 10.1038/sj.bjc.6601240

**Published:** 2003-09-30

**Authors:** A Houston, M W Bennett, G C O'Sullivan, F Shanahan, J O'Connell

**Affiliations:** 1 Department of Medicine, Clinical Sciences Building, National University of Ireland, University Hospital, Cork, Ireland; 2 Department of Surgery, Mercy Hospital, National University of Ireland, University Hospital, Cork, Ireland

**Keywords:** fas ligand, immune privilege, neutrophils, transforming growth factor-*β*, apoptosis

## Abstract

Many cancers express Fas ligand (FasL/CD95L) *in vivo*, and can kill lymphoid cells by Fas-mediated apoptosis *in vitro*. However, overexpression of recombinant FasL in murine tumour allografts revealed a potential antitumour effect of FasL, via recruitment of neutrophils. Transforming growth factor-*β*1 (TGF-*β*1) could inhibit these neutrophil-stimulatory effects of FasL. In the present study, we sought to determine directly whether FasL contributes to immune privilege or tumour rejection in human colon cancers *in vivo*, and whether TGF-*β*1 regulates FasL function. Serial tumour sections were immunostained for FasL and TGF-*β*1. Neutrophils and tumour infiltrating lymphocytes (TILs) were detected by immunohistochemistry for lactoferrin and CD45, respectively. Apoptotic TIL were identified by dual staining for TUNEL/CD45. FasL expression by nests of tumour cells was associated with a mean four-fold depletion of TILs (range 1.8–33-fold, *n*=16, *P*<0.001), together with a two-fold increase in TIL apoptosis (range 1.6–2.5-fold, *n*=14, *P*<0.001), relative to FasL-negative nests within the same tumours. The overall level of neutrophils present in all tumours examined was low (mean 0.3%, *n*=16), with FasL expression by tumour nests associated with a mean two-fold decrease in neutrophils, irrespective of TGF-*β*1 expression. Together, our results suggest that tumour-expressed FasL is inhibitory rather than stimulatory towards antitumour immune responses.

Colorectal cancer represents a formidable health care problem, being one of the major causes of cancer death (Greenlee *et al*, 2001). Despite infiltration of colonic carcinoma tissue with lymphocytes, the immune system fails to effectively eliminate these malignant cells. A number of immune escape mechanisms have been identified, ranging from defective antigen processing and presentation (Kaklamanis *et al*, 1995; Johnsen *et al*, 1999) to the production of immunosuppressive factors (O'Mahony *et al*, 1993; Luo *et al*, 1997). Recent studies have identified an additional mechanism of tumour immune evasion involving expression of the apoptosis-inducing protein Fas ligand (FasL/CD95L) (O'Connell *et al*, 1996). FasL is a member of the TNF family, which can trigger apoptotic cell death following ligation to its receptor, Fas (CD95/APO-1) (Nagata and Golstein, 1995). This interaction plays an important role in immune homeostasis (Nagata, 1999) and in the maintenance of immune privilege in certain organs such as the eye (Griffith *et al*, 1995). The discovery of FasL expression by a variety of tumour cells (Bennett *et al*, 1998; O'Connell *et al*, 1998; Mann *et al*, 1999) raised the possibility that FasL may also mediate immune privilege in human tumours. Tumour infiltrating lymphocytes (TILs) express Fas (Cardi *et al*, 1998) and are sensitive to Fas-mediated apoptosis. In contrast, many human tumours have been shown to be resistant to FasL-induced cell death (O'Connell *et al*, 2000). Thus, upregulation of FasL expression would allow tumour cells to kill infiltrating antitumour lymphocytes. Several studies have shown that tumour-expressed FasL is capable of killing Fas-bearing, sensitive cells *in vitro* (O'Connell *et al*, 1996; Yoong *et al*, 1999), while expression of FasL by human tumours *in vivo* is associated with apoptosis and loss of TILs (Bennett *et al*, 1998). Thus, FasL expression by human tumours may enable the tumour cells to counterattack antitumour effector cells with one of their own apoptosis triggers: FasL.

Based on the ability of FasL to eliminate activated, Fas^+^ T cells, it was hoped that forced expression of FasL would protect allografts from lymphocyte-mediated effector mechanisms. However, rodent transplantation experiments involving FasL have yielded conflicting results. Several groups demonstrated enhanced allograft survival following direct transfection of FasL cDNA into allografts (Li *et al*, 1998; Tourneur *et al*, 2001). Cotransplantation of FasL-negative allografts with FasL-expressing cells was also reported to prevent allograft rejection (Korbutt *et al*, 1997; Takeda *et al*, 1998). This contrasts with other studies where genetically engineered overexpression of FasL resulted in inflammation and allograft rejection (Allison *et al*, 1997; Seino *et al*, 1997a). Rejection was characterised by massive neutrophil infiltration and accelerated allograft destruction. Cotransfection of the immunosuppressive cytokine transforming growth factor-*β*1 (TGF-*β*1) with FasL was sufficient to inhibit the neutrophil-stimulatory activity of FasL, resulting in allograft survival (Chen *et al*, 1998). These findings raise the question as to whether endogenous expression of FasL by human tumours, instead of mediating immune privilege, is in fact detrimental to the survival of the tumour due to neutrophil recruitment. In particular, does TGF-*β*1 determine FasL activity *in vivo*, such that FasL mediates tumour immune privilege where TGF-*β*1 is coexpressed, but recruits neutrophils in its absence?

In the present study, we sought to determine directly if FasL expression by nests of colon tumour cells was associated with increased apoptosis of TILs, thereby contributing to the immune privilege of the tumour. We ascertained the level of neutrophil infiltration in the same colon tumours, and determined whether there was any local recruitment of neutrophils into FasL-expressing tumour nests. We also determined whether the level of neutrophil infiltration into FasL-positive tumour nests was affected by the presence or absence of tumour-expressed TGF-*β*1.

## METHODS

### Tissues

Human colonic adenocarcinomas (*n*=16) were collected following surgical resection at the Mercy Hospital, Cork, following a protocol approved by the University Teaching Hospitals Ethics Committee. None of the patients had received chemo-, radio- or immunotherapy prior to tissue collection. Specimens included tumours of all Dukes' stages: A (*n*=2), B (*n*=7), C (*n*=4) and D (*n*=3).

### Immunohistochemical detection of FasL and TGF-*β* protein

Formalin-fixed, paraffin-embedded colonic tumour sections were deparaffinised in xylene and rehydrated prior to analysis. Antigen retrieval was performed by microwave irradiation in 0.01 M citrate buffer, pH 6.0. Slides were washed with TBS containing 0.001% saponin, and endogenous peroxidase was quenched with 3.0% hydrogen peroxide in methanol for 5 min. Nonspecific binding was blocked with 5% NGS in wash buffer for 1 h. To detect FasL expression, adjacent sections were incubated overnight at 4°C with two different anti-FasL antibodies − rabbit polyclonal anti-human FasL-specific IgG (C-20; Santa Cruz Biotechnology, Santa Cruz, CA, USA) at 0.2 *μ*g ml^−1^ and FasL-specific monoclonal antibody (clone G247-4; PharMingen, San Diego, CA, USA) at 5 *μ*g ml^−1^. Antibody binding was localised using a biotinylated secondary antibody, avidin-conjugated HRP and DAB substrate, contained within the Vectastain ABC detection kit (Vector Laboratories, Burlingame, CA, USA). Slides were counterstained with haematoxylin. The immunising peptide was included at 2 *μ*g ml^−1^ during primary antibody incubation in control staining for the Santa Cruz antibody, while an isotype-matched control in place of the primary antibody was used for the monoclonal antibody.

Transforming growth factor-*β*1 expression was detected in a similar fashion except that antigen retrieval involved treating the slides with hyaluronidase (1 mg ml^−1^) for 30 min at room temperature. TGF-*β*1 protein was detected using a polyclonal anti-human TGF-*β*1-specific IgG (R&D Systems, UK) at 1 *μ*g ml^−1^.

### Immunohistochemical detection of leucocytes (CD45) and neutrophils (lactoferrin)

Tumour-infiltrating immunocytes and neutrophils were identified by immunostaining for CD45 (leucocyte common antigen) and lactoferrin, respectively. Following deparaffinisation and rehydration, antigen retrieval was performed by microwave irradiation in 0.01 M citrate buffer, pH 6.0. The slides were incubated with mouse anti-human CD45 monoclonal IgG (Dako Corp., Carpinteria, CA, USA) diluted 1 : 70, or mouse anti-human lactoferrin monoclonal IgG (PharMingen) at 10 *μ*g ml^−1^ for 1 h. Antibody binding was detected using a secondary rabbit anti-mouse IgG (Dako Corp.) at a dilution of 1 : 25, followed by alkaline phosphatase-conjugated anti-alkaline phosphatase (APAAP) complex (Dako Corp.) at a dilution of 1 : 50. Sections were then incubated for 10 min, with an alkaline phosphatase substrate solution (Fast Red; Sigma Chemical Co., St. Louis, MO, USA). Positive cells appeared red when viewed under light microscopy.

### Detection of apoptotic TILs by CD45/TUNEL dual staining

CD45/TUNEL dual staining was performed to allow apoptotic TILs to be identified and enumerated within the colonic tumours. CD45 staining was performed first on colonic tumour sections as described, except that the colour substrate used was Fast Blue (Sigma Chemical Co.). Cell death was then detected *in situ* by enzymatic labelling of DNA strand breaks using terminal deoxynucleotidyl transferase-mediated dUTP nick end labelling (TUNEL) (Roche Molecular Biochemicals, Indianapolis, IN, USA), according to the manufacturer's instructions. When viewed under light microscopy, nonapoptotic CD45-single-positive cells stained blue, while apoptotic CD45/TUNEL dual-positive cells exhibited brown nuclear staining with blue cytoplasmic/cell surface staining.

### Cell counting and labelling indices

To quantify CD45-positive TIL infiltration of FasL-positive *vs* FasL-negative tumour nests, stained tumour sections were analysed under light microscopy as previously described (Bennett *et al*, 1998). Briefly, FasL-positive and FasL-negative areas were located on a FasL-stained colonic tumour section by one investigator. A consecutive, CD45-stained slide from the same tumour was superimposed on the FasL-stained slide. Using histological landmarks, the corresponding FasL-positive and FasL-negative areas were located on this slide. The FasL-stained section was removed, and a second investigator, blinded as to the local status of FasL expression, counted the number of CD45-positive TIL per 2000 total nuclei in the area located by the first investigator. A similar approach was employed to enumerate CD45/TUNEL dual-positive TIL, and lactoferrin-positive neutrophils within FasL-positive *vs* FasL-negative areas of colonic tumours. For each type of staining, slides from all tumour specimens were stained in a single experiment.

Labelling indices for TIL infiltration and neutrophil recruitment were expressed as the percentage CD45-positive or the percentage lactoferrin-positive cells per 2000 total nuclei counted. Labelling indices for TIL apoptosis were expressed as the percentage CD45/TUNEL dual-positive cells per 500 total CD45-positive cells counted.

## RESULTS

### Local expression of FasL by nests of colon tumour cells is associated with reduced TIL infiltration

**Figure 1:**
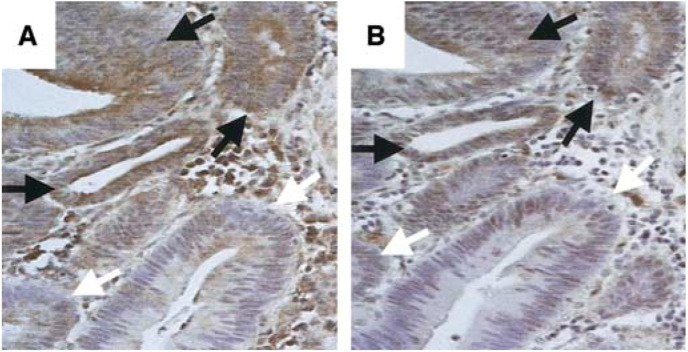
Human colon adenocarcinomas express FasL. FasL expression on consecutive tumour sections was determined by immunoperoxidase staining, using two different antibodies – (**A**) rabbit polyclonal (C-20; Santa Cruz Biotechnology) and (**B**) monoclonal (clone G247-4; PharMingen) FasL-specific antibody. FasL-expressing (brown) tumour nests (black arrows) could be identified adjacent to FasL-negative tumour nests (white arrows) within each colonic adenocarcinoma. There was a good agreement between the immunohistochemical staining pattern obtained with both antibodies, confirming the specificity of FasL detection. Original magnification: × 200.

Using a FasL-specific rabbit polyclonal antibody, FasL was found to be expressed on tumour cells from 16 surgically resected colon cancers, as revealed by intense immunohistochemical staining. Staining within individual tumours varied in intensity and extent, with FasL-positive and FasL-negative neoplastic regions coexisting within all tumours. FasL expression by some TILs was also detected in all specimens. Since questions have been raised regarding the specificity of some anti-FasL antibodies (Stokes *et al*, 1998), FasL expression was confirmed by immunohistochemistry on consecutive tumour sections using two different FasL-specific antibodies (clone G247-4, PharMingen; clone C20, Santa Cruz Biotechnology). There was good agreement between the results obtained with the two antibodies; the pattern of FasL detection corresponded exactly between the consecutive sections (Figure 1

).

**Figure 2:**
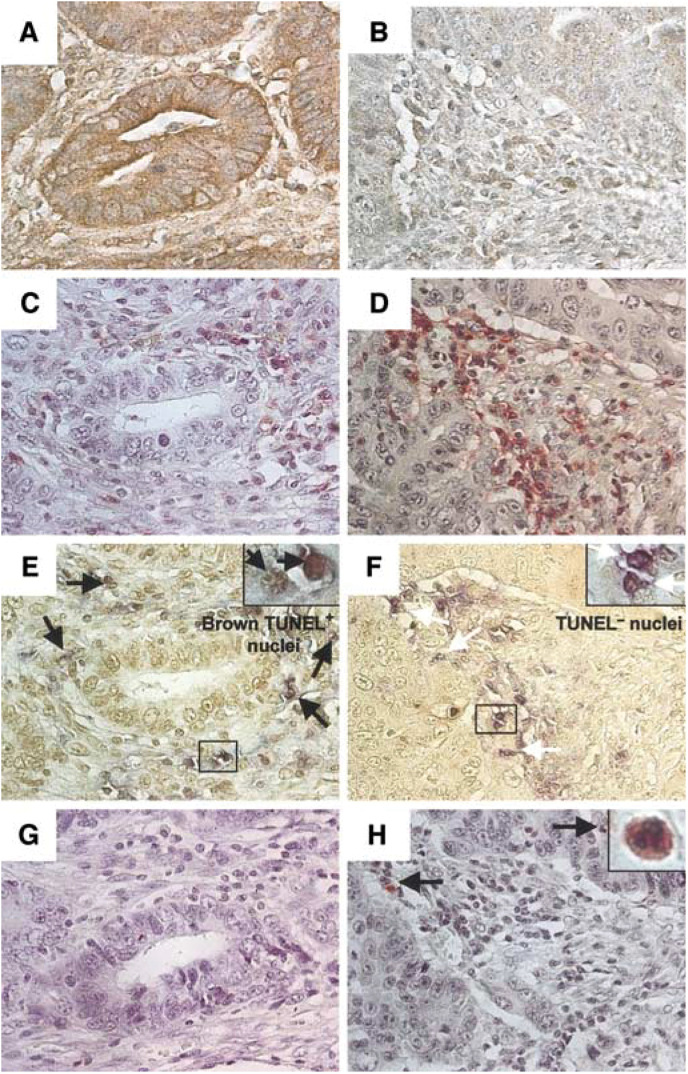
Increased apoptosis leading to depletion of TILs in FasL-expressing nests of colonic adenocarcinomas. **A–F**, FasL-specific immunohistochemical staining (brown) allowed identification of (**A**) FasL-expressing and (**B**) FasL-negative tumour nests in close proximity within individual colonic adenocarcinomas. On a consecutive tumour section, TILs were identified by immunohistochemical staining for CD45 (red). Significantly fewer TILs were evident in tumour nests that expressed FasL (**C**) relative to nests that were negative for FasL (**D**). Tumour-infiltrating lymphocytes that were undergoing apoptosis were identified by CD45–TUNEL dual staining on another consecutive section (**E** and **F**). Apoptotic TIL exhibited brown TUNEL-positive nuclear staining with blue CD45-positive cytoplasmic/cell surface staining (black arrows, **E**). Apoptosis was significantly less frequent among TILs within FasL-negative tumour nests (**F**), where a high proportion of TILs showed absence of TUNEL staining (white arrows), against a blue CD45-positive cytoplasmic background (inset **F**). FasL expressed in human colon cancer does not recruit neutrophils. **G,H**, Neutrophils were identified by immunohistochemical staining for lactoferrin (red), together with their multi-lobed nucleus (inset **H**). There was no significant difference in the level of neutrophil infiltration between colon tumour nests that expressed FasL (**G**) and tumour nests that were negative for FasL (**H**). Original magnifications: × 400 (**A–H**) and × 1000 (insets, **E**, **F** and **H**). The results shown are representative of 16 colonic adenocarcinomas.

**Figure 3:**
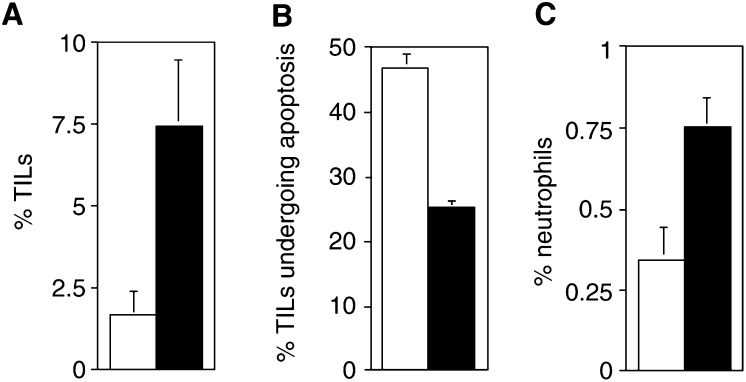
Local expression of FasL by colon tumour nests causes the apoptotic depletion of TILs. (**A–B**), TILs were quantified as percentage CD45-positive cells per 2000 total nuclei counted. There were four-fold fewer TILs detectable within tumour nests that expressed FasL (open bar) compared with nests that did not express FasL (solid bar) (range 1.8–33-fold, *n*=16, *P*<0.001; Wilcoxon signed rank) (**A**). TILs undergoing apoptosis were quantified as percentage TUNEL-CD45 dual positive cells per 500 total CD45-positive cells detected. There was consistently two-fold more apoptosis of TILs within tumour nests that expressed FasL (open bar) compared with nests that did not express FasL (solid bar) in each tumour (range 1.6–2.5-fold, *n*=14, *P*<0.001; Wilcoxon signed rank) (**B**). Local expression of FasL by tumour nests does not recruit neutrophils into colon cancers. (**C**), Intratumoral neutrophils were quantified as percentage lactoferrin-positive cells per 2000 total nuclei counted. The overall level of neutrophils was low (mean=0.3%, *n*=16). There was no recruitment of neutrophils into tumour nests that expressed FasL. In contrast, there were more than twofold fewer neutrophils present in tumour nests that expressed FasL (open bar) relative to nests that did not (solid bar) (**C**), although this difference in neutrophil infiltration was not statistically significant.

Leucocyte infiltration in tumours was assessed by immunohistochemical staining for the leucocyte common antigen (CD45). Although all tumours exhibited infiltration by lymphocytes, within individual tumours there was marked regional variation in the numbers of infiltrating lymphocytes. To evaluate whether FasL expression by colon tumour cells affected the level of TILs, the number of CD45-positive TILs within FasL-positive *vs* FasL-negative tumour cell nests was assessed. Using a reference section immunohistochemically stained for FasL to identify FasL-positive and FasL-negative tumour nests, we found that there were consistently fewer TILs within FasL-expressing nests relative to FasL-negative tumour nests within each tumour examined (*n*=16) (Figure 2

). Results revealed that there was a mean four-fold fewer TILs in tumour nests that expressed FasL relative to those nests that did not express FasL (range 1.8–33-fold, *n*=16, *P*<0.001; Wilcoxon signed rank) (Figure 3A

). This indicated that FasL expression by nests of colon tumour cells appeared to have an inhibitory effect on infiltration by TILs.

### FasL expression in colon tumour nests is associated with increased apoptosis of TILs

To investigate if the impaired lymphocyte infiltration into FasL-expressing colon tumour nests was due to apoptotic depletion, we performed dual staining combining TUNEL with immunohistochemistry for CD45. Using reference sections immunohistochemically stained for FasL, intratumoral variation in the level of TIL apoptosis was observed, with consistently more apoptosis of TILs in FasL-expressing tumour nests (Figure 2). Significantly, there were consistently two-fold more apoptotic TILs within tumour nests that expressed FasL relative to those nests that did not (Figure 3B) (range 1.6–2.5-fold, *n*=14, *P*<0.001; Wilcoxon signed rank) (*n*=14; two of the 16 tumours did not contain sufficient FasL-negative nests to reach a count of 500 TILs). Together, these results demonstrate that FasL expression by colon tumour nests is associated with apoptosis of CD45-positive TILs, resulting in depletion of TILs within these tumour nests.

### FasL expression in colon cancer is not associated with recruitment of neutrophils

Given the recent findings that transplantation of allografts genetically engineered to express FasL may result in neutrophil recruitment and graft rejection, we sought to determine if endogenous FasL expressed by colon tumour nests was proinflammatory and responsible for any local recruitment of neutrophils. The extent of neutrophil recruitment was determined by counting the number of neutrophils within adjacent FasL-positive and FasL-negative tumour nests. Neutrophils were identified based on their characteristic multi-lobed nuclei, together with immunohistochemical staining for the neutrophil marker lactoferrin (Figure 2). The overall level of neutrophils present within the tumour specimens was low (mean=0.3%, *n*=16) (Table 1

**Table 1:** Lack of neutrophil recruitment in response to colon tumour-expressed FasL, irrespective of TGF-*β*1 expression

			**% Neutrophils**†	
**Tumor specimen**	**FasL Expression**‡	**TGF-*β*1 Expression**†	**FasL^+^ Tumor**	**FasL^−^ Region**
1	+++	++	0.55	0.85
2	+++	++	0.15	1.0
3	+++	−	0.5	0.05
4	++++	++++	0.25	0.0
5	++	+++	0.7	1.95
6	++++	+	0.0	0.05
7	++++	−	0.0	0.0
8	+++	++++	0.2	0.35
9	+++	−	0.0	0.0
10	+++	−	0.05	0.0
11	++++	+	0.05	0.3
12	++++	+	0.2	0.85
13	+++	−	0.2	0.0
14	++++	−	0.15	0.5
15	++++	+++	0.0	0.15
16	++	−	0.1	0.0

† Based on % positive cells: +=1−25%; ++=26−50%; +++=51−75%; ++++=76−100%.

‡ % neutrophil recruitment was calculated as the % lactoferrin-positive cells per 2000 total nuclei counted within FasL-positive/FasL-negative tumour regions.

) In particular, there was a lack of neutrophil recruitment in response to tumour-expressed FasL. There were two-fold fewer neutrophils in tumour nests that expressed FasL compared to nests that did not express FasL (*n*=16) (Figure 3C). Activated neutrophils have previously been shown to be sensitive to Fas-mediated apoptosis *in vitro* (Liles *et al*, 1996). Our findings are consistent with a role for colon tumour-expressed FasL in causing apoptosis rather than activation or recruitment of neutrophils.

### Lack of FasL-mediated recruitment of neutrophils in colon cancer is not dependent on expression of TGF-*β*1

**Figure 4:**
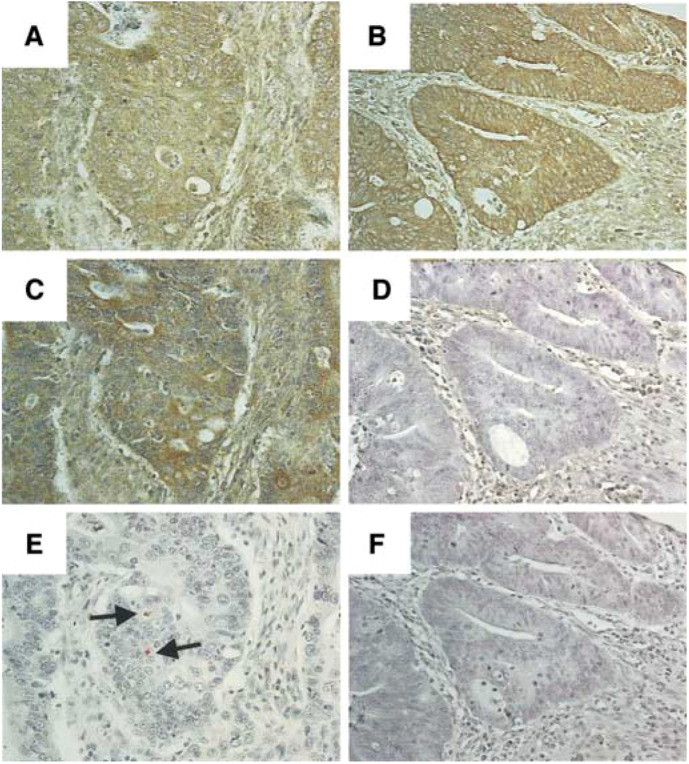
FasL expression by colonic tumour cells does not mediate neutrophil recruitment, irrespective of TGF-*β*1 expression. (**A–F**), FasL-expressing tumour cell nests (brown) were identified by immunohistochemistry (**A** and **B**). On a consecutive tumour section, TGF-*β*1-specific immunoperoxidase staining (brown) allowed the TGF-*β*1 status of the colonic tumour cell nests to be identified. FasL-expressing colon tumour nests that coexpressed (**C**) and failed to express (**D**) TGF-*β*1 could be identified. Neutrophils were identified by immunohistochemical staining for lactoferrin (red, arrows) on an adjacent section. The overall level of neutrophil infiltration was low, irrespective of FasL expression, or of the presence (**E**) or absence (**F**) of TGF-*β*1. Original magnifications: X 400 (**A***–***f**). The results shown are representative of 16 colonic adenocarcinomas.

**Figure 5:**
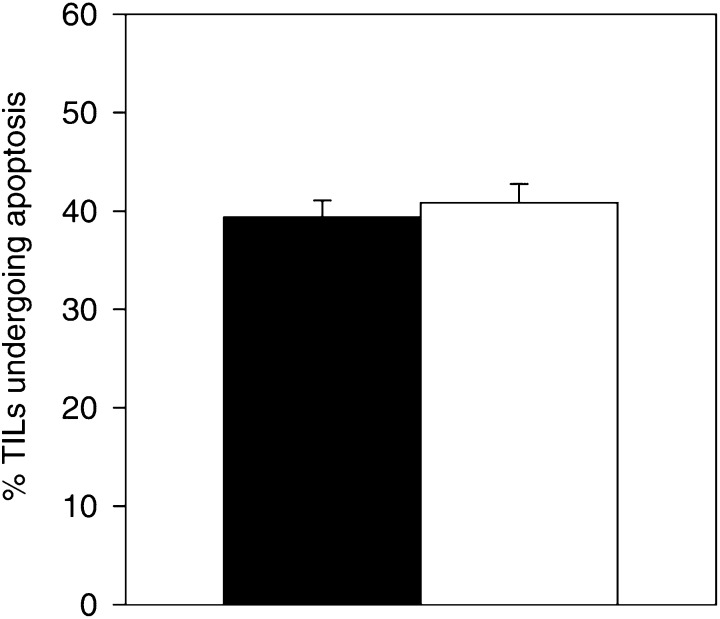
Expression of TGF-*β*1 by colon tumours does not correlate with the overall rate of TIL apoptosis. Apoptotic TILs were quantified as the percentage TUNEL–CD45 dual positive cells per 500 total CD45-positive cells counted. There was no significant difference between the overall percentage of TILs undergoing apoptosis in tumours that expressed TGF-*β*1 (solid bar) relative to tumours that did not express TGF-*β*1 (open bar) (*n*=14, *P*=0.282; Mann-Whitney *U* test).

Since TGF-*β*1 has been shown to be capable of controlling the proinflammatory effects of FasL overexpression (Chen *et al*, 1998), we sought to determine if expression of TGF-*β*1 might account for the lack of neutrophil recruitment in FasL-positive colon cancers. Immunohistochemical staining identified TGF-*β*1-producing neoplastic cells within our panel of colon adenocarcinomas. The expression of TGF-*β*1 was variable in the tumours (*n*=16): four were strongly positive, five were moderate or weakly positive, and seven were negative (Table 1). The seven tumours that were completely negative for TGF-*β*1 were strongly positive for FasL, yet the overall level of neutrophil infiltration in these tumours was low, and was not higher than in tumours that coexpressed FasL and TGF-*β*1. Examination of individual tumour sections revealed that there was no local recruitment of neutrophils into tumour nests that were positive for FasL and negative for TGF-*β*1, or into nests that coexpressed FasL and TGF-*β*1 (Figure 4

). Hence, tumour-expressed FasL is not associated with general or local neutrophil recruitment in the tumour microenvironment. This is irrespective of whether TGF-*β*1 is coexpressed or not. Moreover, the increased apoptosis of TILs found in FasL-expressing tumour nests was not dependent on TGF-*β*1 expression. The overall apoptotic rates of TILs in TGF-*β*1-expressing tumours (mean=33.81%) and TGF-*β*1-negative tumours (mean=35.67%) were not significantly different (*P*=0.282; Mann–Whitney *U* test) (Figure 5

).

## DISCUSSION

Despite compelling evidence demonstrating an immune evasive function for tumour-expressed FasL, controversy surrounds this protein (O'Connell *et al*, 2001; Restifo, 2000), with some reports suggesting that FasL expression by tumour cells is proinflammatory and accelerates tumour elimination. Thus, in the present study, the ability of colon tumour-expressed FasL to eliminate TILs and to contribute to local immune suppression was analysed directly *in situ*. The expression of FasL by human colon cancer cells is well established. Using multiple techniques, including *in situ* hybridisation for FasL mRNA and immunodetection for FasL protein using a variety of different FasL-specific antibodies, FasL expression by colon cancer has been demonstrated both *in vivo* and *in vitro* (Shiraki *et al*, 1997; O'Connell *et al*, 1998; Mann *et al*, 1999; Yoong *et al*, 1999). Furthermore, several studies have demonstrated the ability of colon tumour cells to kill Fas-sensitive target cells *in vitro*. FasL-expressing colon cancer cells have been shown to kill both co-cultured Fas-sensitive Jurkat T cells (O'Connell *et al*, 1996; Shiraki *et al*, 1997) and primary hepatocytes (Yoong *et al*, 1999). FasL-specific antisense oligonucleotide treatment of the SW620 cells, which transiently inhibited their ability to express FasL, protected the Jurkat cells from FasL-mediated killing (O'Connell *et al*, 1996). We confirmed FasL expression by human colon adenocarcinomas by immunohistochemistry using two different FasL-specific antibodies. The examination of consecutively stained sections revealed an identical immunohistochemical staining pattern with both antibodies, confirming the specificity of FasL detection.

The expression of FasL by the tumour cells was found to vary greatly within individual tumours. Since both FasL-positive and FasL-negative regions coexisted within individual tumours, a direct analysis of the ability of tumour-expressed FasL to induce apoptosis of infiltrating lymphocytes could be performed. In the present study, FasL expression by nests of colon tumour cells was found to be significantly and consistently associated with increased apoptosis of TILs, leading to dramatically diminished infiltration of TILs into these areas of the tumours. There was a consistent (mean four-fold) decrease in TILs in FasL^+^ relative to FasL^−^ tumour islands, concomitant with a consistent (mean two-fold) increase in TIL apoptosis. Since these results are from matched FasL-positive and FasL-negative tumour islands within the same individual tumour specimens, the apoptosis and depletion of tumour infiltrating lymphocytes is unlikely to be due to factors other than tumour-expressed FasL. Tumour infiltrating lymphocytes express Fas (Cardi *et al*, 1998) and activated T cells are sensitive to Fas-mediated apoptosis (Krammer, 1999). Thus, these results support a role for FasL in mediating immune privilege in human colon tumours, via the apoptotic depletion of infiltrating antitumour lymphocytes. The ability of colon tumour cells to counterattack the antitumour immune response may provide malignant cells with a potent mechanism with which to evade the immune system, promoting the survival and progression of the tumour.

FasL has recently been shown in rodent transplantation studies to possess proinflammatory activity (Allison *et al*, 1997; Seino *et al*, 1997a), suggesting that expression of this protein, instead of protecting tumour cells from the immune response, could in fact be detrimental to tumour survival. Inflammation was characterised by neutrophil recruitment and allograft destruction. Examination of our tumour specimens revealed that FasL expression by colon tumour cells is not proinflammatory. Analysis of matched FasL-positive and FasL-negative tumour nests revealed that local expression of FasL by tumour cells is not associated with neutrophil recruitment. Not only was there a lack of neutrophil infiltration, but in fact there were two-fold fewer neutrophils in tumour nests that expressed FasL relative to those that did not. Neutrophils express Fas and are sensitive to Fas-mediated apoptosis *in vitro* (Liles *et al*, 1996), and so perhaps are susceptible to killing by tumour-expressed FasL *in vivo*, further impairing the immune response. Moreover, all studies demonstrating inflammation and neutrophil recruitment involve forced overexpression of FasL (Allison *et al*, 1997; Kang *et al*, 1997; Seino *et al*, 1997b). There are no studies demonstrating FasL-dependent neutrophil recruitment in response to native FasL. In contrast, under physiological conditions, native FasL is consistently associated with apoptosis of activated lymphocytes and immune privilege (Bellgrau *et al*, 1995; Bennett *et al*, 1998). In agreement with this, our results clearly demonstrate that upregulation of FasL expression during human colon carcinogenesis does not trigger neutrophil recruitment.

Recently, TGF-*β* has been shown in experimental rodent allograft studies to suppress the proinflammatory activity of overexpressed FasL. Transformine growth factor-*β*1 is an immunosuppressive cytokine, which inhibits the proliferation and activity of T cells and NK cells (Letterio and Roberts, 1998). Using our panel of human colon tumours, we found that lack of neutrophil recruitment in response to tumour-expressed FasL was independent of TGF-*β*1 production by the tumour cells. Transformine growth factor-*β*1 was produced with varying extent by 56% (nine out of 16) of the tumours. Analysis of individual tumours revealed a lack of neutrophils in FasL-positive tumour nests that failed to coexpress TGF-*β*1. In fact, seven of the tumours did not produce TGF-*β*1 but expressed FasL, without the presence of a neutrophilic infiltrate. Furthermore, the apoptotic depletion of TILs found in FasL-expressing colon tumour nests relative to FasL-negative tumour nests was independent of TGF-*β*1 expression by the tumour cells. Evidence from studies of TGF-*β*1^−/−^ mice suggests that TGF-*β*1 plays a role in regulating lymphocyte homeostasis in the periphery, in part by exerting an inhibitory effect on lymphocyte apoptosis (Bommireddy *et al*, 2003). Examination of our tumour specimens revealed that expression of TGF-*β*1 by the tumour cells did not correlate with the overall apoptosis rate of TILs. Our findings indicate that upregulation of FasL expression in tumours appears to promote the survival of the neoplastic cells via apoptotic depletion of TILs, irrespective of the presence of the immunosuppressive cytokine TGF-*β*. Establishment of immune privilege is a multifactorial process. In the eye, FasL and TGF-*β* represent only two of at least eight independent mechanisms that function to preclude any potentially destructive inflammatory responses (Ferguson and Green, 2001). Similarly, the immune downregulatory activity of FasL may be favoured by a combination of immunosuppressive processes in the tumour microenvironment. Selective pressure during tumour development would ensure that FasL upregulation would only occur where it would be advantageous to the tumour.

Together, our results show that FasL contributes to immune privilege in human colon cancer *in vivo* via apoptotic depletion of TILs. Despite strong expression of FasL in each specimen, the overall level of neutrophils present in all of the tumours was low. Absence of any FasL-induced inflammation was not dependent on TGF-*β* coexpression. Thus, upregulation of FasL expression during colon carcinogenesis is advantageous rather than detrimental to tumour survival. In fact upregulation of FasL expression by colonic tumour cells has been shown to occur early on in the pathogenesis of this malignant disease (Bennett *et al*, 2001; Shimoyama *et al*, 2001), suggesting that expression of FasL may be a fundamentally important and even necessary event in the transformation process. Functional T cells are central to an intact antitumour immune response, and so elimination of TILs by apoptosis in response to tumour-expressed FasL represents a potent mechanism of tumour immune evasion.
